# Adequate initial antimicrobial therapy as the factor assessing treatment efficacy in human septic shock

**DOI:** 10.1186/cc14185

**Published:** 2015-03-16

**Authors:** P Szturz, P Folwarczny, J Švancara, R Kula, P Ševèík

**Affiliations:** 1 University Hospital and Faculty of Medicine Ostrava University, Ostrava, Czech Republic; 2 Institute of Biostatistic and Analyses, Masaryk University, Brno, Czech Republic

## Introduction

The early identification of severe sepsis and septic shock and early implementation of the SSC bundles were associated with reduced mortality [1]. The failure to initiate appropriate antimicrobial therapy increased mortality of septic shock patients [2]. We hypothesized that the parameter 'Consensus initial antimicrobial therapy with microbial cultures' correlates with outcome of septic shock patients.

## Methods

We analyzed 535 consecutive patients with septic shock (sepsis-induced hypotension persisting despite adequate fluid resuscitation) from the EPOSS database (Data-based Evaluation and Prediction of Outcome in Severe Sepsis), which was developed to monitor and assess treatment efficacy in patient with severe sepsis and septic shock. Patients were admitted to participating ICUs (12 hospitals - 17 high-volume care units) in the Czech Republic from 1 January 2011 to 5 November 2013. Patients were divided into two groups: survivors (*n *= 274) and nonsurvivors (*n *= 261).

## Results

Survivors versus nonsurvivors were similar in: age 65.8 (64.2; 67.5) versus 66.5 (64.7; 68.3) *P *= 0.583, men 159 (58.0%) versus 160 (62.0%) *P *= 0.376, APACHE II score 27 (15 to 40) versus 28 (15 to 40) *P *= 0.737. Statistically significant differences between survivors versus nonsurvivors were found in the parameter 'Consensus initial antimicrobial therapy with microbial cultures' 178 (79.5%) versus 128 (58.4%) *P *< 0.001 and in the parameter 'Administration antimicrobials within the first hour' 163 (59.9%) versus 171 (70.7%) *P *= 0.001. Administration of 30 ml/kg crystalloid for hypotension or lactate 4 mmol/l (3 hours) and application of vasopressors (6 hours) were in both groups without statistically significant differences.

## Conclusion

We found that correct choice of antibiotics improves outcome of septic shock patients. The choice of empirical antimicrobial therapy depends on complex factors related to the underlying disease, susceptibility of pathogens, patient's history and clinical syndrome. Adequate initial antimicrobial therapy as an important factor of survival along with suitable initial fluid resuscitation and application of vasopressors should be a priority for healthcare in human septic shock.
